# Age-dependent shifts and spatial variation in the diet of endangered Black-faced Spoonbill (*Platalea minor*) chicks

**DOI:** 10.1371/journal.pone.0253469

**Published:** 2021-07-09

**Authors:** Min-Su Jeong, Chang-Young Choi, Woo-Shin Lee, Ki-Sup Lee

**Affiliations:** 1 Department of Agriculture, Forestry, Bioresources, Seoul National University, Seoul, Republic of Korea; 2 Research Institute of Agriculture and Life Sciences, Seoul National University, Seoul, Republic of Korea; 3 Waterbird Network Korea, Seoul, Republic of Korea; MARE – Marine and Environmental Sciences Centre, PORTUGAL

## Abstract

The endangered Black-faced Spoonbill (*Platalea minor*) strictly breeds in marine environments and is threatened by the rapid loss of coastal wetlands within its breeding range. Adults with chicks are thought to gradually switch feeding sites from freshwater wetlands to coastal mudflats as the chicks’ osmoregulatory system develops. We investigated age-dependent shifts in the diet of Black-faced Spoonbill chicks at four breeding colonies with varying freshwater habitat availability by examining stable isotopes (δ^13^C, δ^15^N) between the tip (grown at the age of 10 days) and middle (grown at the age of 22 days) portions of their primary feathers. The δ^13^C value of the middle portions was significantly higher than that of the tips, which suggested that the ratio of marine resources increased with the growth and development of chicks. A Bayesian isotope mixing model revealed that the diet proportion of marine prey in the early-chick rearing season was slightly higher than in the late-chick rearing season at three colonies in inshore areas, although this proportion was approximately 60% even in the early chick-rearing period. In contrast, isotopic values and reconstructed diet composition suggested that chicks in an offshore colony with limited freshwater wetlands relied more heavily on freshwater diets for both chick-rearing periods (>80%). Our results suggest that the shifts in feeding sites seen in previous studies might be related to the age-dependent dietary shift of chicks, highlighting the importance of freshwater wetlands for spoonbills on offshore islands without an inflow of freshwater in nearby intertidal mudflats. These findings emphasize the importance of freshwater prey and wetlands even for the endangered marine-breeding spoonbills, even though the negative impact of salt stress remains inconclusive.

## Introduction

Colonial nesting waterbirds are typical central place foragers, repeating trips between the breeding colony and remote foraging areas. Thus, the energetic costs for feeding chicks depend on the distribution and abundance of prey within their foraging range [1]. In many species, spatial and temporal variation in prey availability around the breeding colony leads to differences in diet and foraging strategies within and between populations [2, 3]. Rapidly growing chicks often have different dietary requirements than adults, such as high caloric or more digestible diets, which can also influence the foraging behavior of parents [4–6]. The abundance of high-quality prey for chick-rearing is an important factor in reproductive output, fitness consequences, and, thus, population dynamics [7–9]. Therefore, understanding the use of resources and foraging habitats during the breeding season is essential for developing a conservation strategy for threatened bird species.

The Black-faced Spoonbill (*Platalea minor*) is an endangered wading bird distributed along with coastal areas of East Asia; it has the smallest range of the six *Platalea* species in the family Threskiornithidae [10, 11]. Although its global population has increased in recent decades, rapid loss of habitat due to land reclamation and industrial development around the Yellow Sea [12, 13], a key breeding area for the spoonbill, is still a major threat to this endangered species [11]. Black-faced Spoonbills forage in a wide variety of wetlands from estuaries and intertidal mudflats to shallow freshwater lakes, artificial ponds, and rice paddies [14]. They mainly prey upon fish and crustaceans, but also consume various aquatic organisms, including insects, mollusks, and amphibians [15, 16].

Unlike the other five spoonbill species that may breed both in marine and freshwater wetlands, Black-faced Spoonbills only breed in marine environments, nesting on uninhabited coastal and offshore islets that are found mostly along the western Korean Peninsula [10, 15, 17]. However, according to a survey of adult spoonbills foraging in rice paddies and intertidal mudflats during the breeding season, rice paddy foraging peaked in May, the beginning of the chick-rearing period, and then gradually decreased in June and July [17]. In contrast, the number of adults foraging in intertidal mudflats increased as the chick-rearing season progressed [17]. Other studies on Black-faced Spoonbills breeding on offshore islets reported that the regurgitated diets of chicks primarily comprised species originating from freshwater wetlands, even though intertidal mudflats were available near their breeding colonies [17, 18]. These preliminary findings indicated high reliance on freshwater wetlands by Black-faced Spoonbills during the early chick-rearing season, which was explained by the limited osmoregulation ability of chicks [17, 18].

Waterbirds living in saline habitats can regulate excess salt consumed with prey through salt glands [19–21], which excrete a concentrated solution to reduce osmotic stress from saline diets and maintain ion homeostasis [22]. However, newborn chicks of many waterbirds, such as White Ibises (*Eudocimus albus*) in the same family Threskiornithidae, as well as Common Eiders (*Somateria mollissima*) and Laughing Gulls (*Leucophaeus atricilla*), have little osmoregulatory capacity [23–25]. Previous studies showed that adults might attempt to reduce the salt intake of their chicks by feeding food regurgitate diluted with body fluids or semi-digested food, to prevent dehydration and salt-loading [5, 24, 26]. In addition, Laughing Gulls, White Ibises, and Scarlet Ibises (*E*. *ruber*) in saline habitats provide chicks with low-salinity diets by changing foraging habitats from salt marshes to inland freshwater wetlands after chick hatching [27–31]. Thus, the Black-faced Spoonbill, which breeds in marine habitats, may forage in freshwater wetlands during the early chick-rearing season to avoid osmotic stress among chicks by providing low-salt prey. Alternately, environmental and phenological conditions may cause the observed temporal changes in foraging habitats and prey species in the Black-faced Spoonbill. Prey availability in rice paddies in coastal South Korea increases after irrigation around mid-May due to the spawning of fish and hatching of aquatic larvae. Around mid-June, rice paddies are drained for approximately seven days to improve tillering of rice, which decreases prey availability as fish and invertebrates move to canals and streams [32–36]. In addition to changes in water depth and abundance or composition of prey species in rice paddies, changes in the size and density of growing rice over time may hamper specific feeding strategies, affecting foraging efficiency and feeding rate. These hypothesized changes in foraging habitat or diet selection of breeding Black-faced Spoonbills, however, are based mainly on anecdotal and observational reports conducted in limited study areas [17, 18], and have not been examined in a quantitative manner.

The stable isotope ratio in consumer tissues can provide useful information on such topics because it reflects the stable isotope ratio of diet in a predictable manner [37–39]. Many diet studies commonly use two stable isotopes: carbon and nitrogen. The carbon stable isotope ratio (δ^13^C) provides insight into the relative importance of marine versus freshwater prey in the diets of consumers [40, 41]. Organisms in marine ecosystems have a more enriched δ^13^C value than those in freshwater because of differences in the carbon sources of primary production between the two ecosystems [42, 43]. The nitrogen stable isotope ratio (δ^15^N) is a useful measure of trophic level because consumer tissues are enriched at successive trophic levels [44]. Keratinized tissues such as feathers are metabolically inactive once they are synthesized; thus, they preserve an isotopic composition that reflects diet during feather growth [45]. Several studies have investigated dietary changes during feather development by analyzing stable isotopes in different feather portions of some waterbirds including Northern Fulmars (*Fulmarus glacialis*) [46], Laughing Gulls [27, 47], and Eurasian Spoonbills [2].

In this study, we sought to determine whether the observed change in foraging habitats of breeding Black-faced Spoonbills was related to an age-dependent shift in diets of chicks at four breeding colonies on the west coast of the Korean Peninsula. We compared the δ^13^C and δ^15^N stable isotopes of the tip and middle portions of a primary feather from each chick, reflecting diet at different ages. In addition, prey availability for each breeding spoonbill may vary depending on hatching timing, which may influence foraging behavior. Therefore, we controlled for the possible effect of seasonal variation in foraging habitat characteristics by including estimated hatching dates for each chick as a covariate in statistical analysis. δ^13^C values were expected to be lower in the tip portions than the middle portions if adult spoonbills exhibited a foraging shift from freshwater to intertidal wetlands as their chicks grew. In addition, we compared stable isotope values between breeding colonies to investigate spatial variation in reliance on freshwater wetlands of breeding Black-faced Spoonbills among four breeding populations with varied foraging habitat availability.

## Materials and methods

### Study sites and foraging habitats

**Fig 1:**
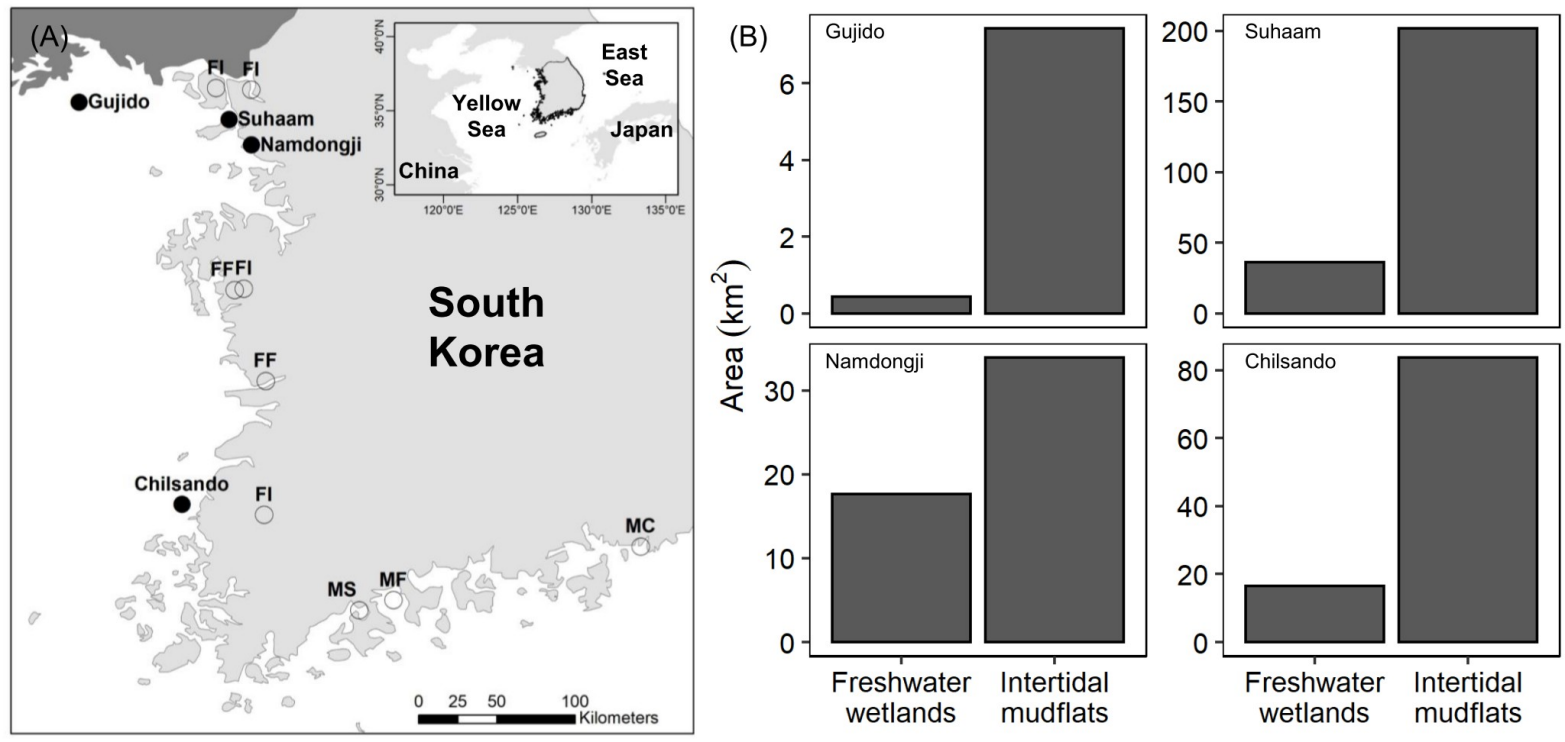
(A) Location of the studied breeding colonies of Black-faced Spoonbills and sampling sites of the potential prey items. (B) Area of the intertidal mudflats and freshwater wetlands within an 11-km radius of each colony. Light and dark gray represent South and North Korea, respectively. Circles represent the sampling sites of potential diets: FF: freshwater fish [49, 50], FI: freshwater invertebrates [51, 52], MF: marine fish [53], MS: marine shrimp [54], and MC: marine crustacean [55]. The base map was creasted using Natural Earth.

This study was conducted at four breeding colonies of Black-faced Spoonbills along the west coast of South Korea (Fig 1). The Gujido Island colony (Gujido; 37.6380°N, 125.6817°E) is located on an offshore island 15 km south of the southwestern part of North Korea. In the coastal areas nearest North Korea, there are intertidal mudflats and rice paddies in landfilled areas [47]. The colony at Suhaam Rock or Islet (Suhaam; 37.5397°N, 126.5428°E) is surrounded by the wide intertidal zone of Incheon City, but there are also freshwater wetlands, such as rivers, streams, and rice paddies, near the colony. The Namdong Reservoir colony (Namdongji; 37.3916°N, 126.6753°E) in Incheon breeds on a tiny artificial islet at a detention reservoir, which was previously an intertidal mudflat but is now separated from the remaining mudflats by a sea wall and a sluice gate. There are freshwater wetlands including streams and rice paddies located ~8 km east of Namdongji [48]. The Chilsando Island colony (Chilsando; 34.1333°N, 125.1167°E) is located on a coastal island 3 km away from the nearest intertidal mudflats and 7 km away from rice paddies along the coast of the Yeonggwang District.

During the chick-rearing period, adult Black-faced Spoonbills forage within an average distance of 11 km around breeding colonies [17]. To determine foraging habitat availability, we estimated the total area of freshwater wetlands and intertidal mudflats within an 11-km radius of each breeding colony using available GIS databases (coastal information maps for intertidal mudflats in 2011 at 1:25,000 scale, Ministry of Maritime and Fisheries; land cover maps for freshwater wetlands in 2013 at 1:5,000 and 1:25,000 scale, Ministry of Environment) and statistics on land use [56] in Korea (Fig 1B). We used the area of freshwater, freshwater wetland, and rice paddies in the land cover maps to estimate the area of freshwater wetlands around Suhaam, Namdongji, and Chilsando. To estimate the area of freshwater wetlands around Gujido, we used the area of rice paddies on inhabited islands near Gujido reported in statistics on land use [56]. All GIS data were processed and analyzed in ArcMap 10.1 [57].

### Sampling procedure

We visited breeding colonies once or twice to collect primary feather samples from June to July 2013. Chicks were captured on or near their nests within the colony. A total of 34 Black-faced Spoonbill chicks were captured by hand or using hand-held nets: six individuals at Gujido, eight individuals at Suhaam, seven individuals at Namdongji, and 13 individuals at Chilsando (Table 1). The captured chicks were measured (length of the wing, tarsus, bill, tail, and head), weighed, and banded with an individually-numbered metal ring as well as a color ring before release. Although the growth rate is still unknown in this species, the Eurasian Spoonbill, which is closely related to the Black-faced Spoonbill [58], has a similar length of wingspan and a chick-rearing period before fledgling [59, 60, 61]. Therefore, we estimated the hatching dates of chicks based on measured wing length using the growth curve for the Eurasian Spoonbill [62] and the sex discriminant function for Black-faced Spoonbill chicks (Table 1) [63].

**Table 1:** The number of Black-faced Spoonbill chicks sampled in each study colony and respective age and hatching date (mean ± SD with a range in parentheses).

Site	No. of sampled individuals	Estimated age	Estimated hatching date (May 1^st^ = 1)
Gujido	6	42.50 ± 7.23 (34–52)	10.80 ± 7.32 (1–20)
Suhaam	8	32.5 ± 3.35 (28–37)	18.1 ± 9.14 (5–29)
Namdongji	7	33.43 ± 2.44 (29–37)	22.6 ± 10.1 (16–51)
Chilsando	13	36.08 ± 5.57 (26–48)	15.5 ± 5.61 (4–26)

We collected approximately 2 cm^2^ feather vanes from the distal tip and the middle part (8 cm from the tip) of the 8th primary feather for stable isotope analysis. The estimated hatching dates of sampled chicks were between May 1 and June 15, and all were more than 25 days old (Table 1) with an 8th primary feather long enough for sampling at the tip and 8 cm from the tip (longer than 10 cm in length). We considered the isotope signatures of the collected primary tips to reflect diets 10 days after hatching [37] because Black-faced Spoonbill chick primaries emerge at 10 days old [64]. As the growth rate of primaries is still unknown in this species, we estimated that the middle part of the primaries represented isotopic values of chick diets at around 22 days (probably 21–23 days) after hatching, based on the average daily growth rate of the primary feathers in the closely related Eurasian Spoonbill (6.6 ± 0.5 mm) [2]. Animal handling and sample collection were approved by the Korean Cultural Heritage Administration (NRICH-1507-B03F-1).

### Stable isotope analysis and Bayesian isotope mixing model

Feather samples were cleaned to remove oil or dirt that could affect the value of the stable isotope ratio by using both a chloroform:methanol 2:1 solution and detergent, as a previous study indicated that cleaning feathers with a solvent might not be sufficient to ensure accuracy and repeatability of isotope analysis [65]. We washed the sampled feathers with a chloroform:methanol 2:1 solution and dried them for a day under a fume hood. Then, the sampled feathers were cleaned with detergent (Deconex 16 plus; Borer Chemie, Zuchwil, Switzerland), rinsed three times with distilled water, and dried under the fume hood [65]. The washed samples were cut and homogenized with stainless steel scissors. δ^13^C and δ^15^N analyses were conducted using a continuous-flow stable isotope ratio spectrometer (IsoPrime-EA, Micromass, Manchester, UK) linked with a CN analyzer (NA Series 2, CE Instruments, Wigan, UK) at the National Instrumentation Center for Environmental Management (NICEM) at Seoul National University of the Republic of Korea.

Stable isotope ratios were expressed in δ-values and parts per thousand (‰), according to the following equation:

$$\delta X=[(R_{sample}/R_{standard})-1]\times 1000$$

where *X* represents δ^13^C and δ^15^N, *R* is the corresponding ratio ^13^C/^12^C and ^15^N/^14^N, and *R*_*standard*_ is the ratio of the international references: Vienna Peedee Belemnite (VPDB) for carbon and air for nitrogen. The known measurement precision with the reference materials in NICEM was better than 0.2‰ for δ^13^C (IAEA-C5, δ^13^C = -25.5‰) and 0.2‰ for δ^15^N (IAEA-N2, δ^15^N = +20.3‰), respectively [66, 67].

The Bayesian isotopic mixing model was used to estimate the relative proportions of the prey for each group by age and breeding colonies with the MixSIAR package [68] and R software version 4.0.2 [69]. No δ^15^N values were obtained for the distal portion (early chick-rearing period) in three individual chicks from Chilsando due to technical problems and sample loss during analysis. We replaced the missing data with mean δ^15^N values of a distal portion of feathers from chicks in the early chick-rearing period in Chilsando. We ran the model with Markov chain Monte Carlo sampling based on the following parameters: number of chains = 3; chain length = 300,000; and thin = 100. We examined model convergence with a Gelmin-Subin Heidelberger diagnostic test and trace plots. To include the main diet groups of the spoonbill, we used the isotopic values of potential prey including fish, crustaceans, and aquatic insect larvae collected from freshwater and intertidal habitats in South Korea reported in previous studies (Fig 1A, Table 2). We primarily included the species of taxa recognized as prey of spoonbills during the breeding season through the analysis of feces and regurgitates and observed foraging behavior in the northwest coastal region where Suhaam and Namdongji are located [17, 18]. We also used the isotopic values of species *Metapenaeopsis dalei* and *Callianassa japonica*, collected in intertidal mudflats along with reported prey species of spoonbills. We checked that all species of fish and crustaceans were distributed throughout the Korean Peninsula or west coast based on a description of habitat and distribution provided in the database of the National Institute of Biological Resources [70]. Insect larvae were collected in rice paddies located along the west coast of South Korea. We assumed that all species could be available to the four breeding populations. We categorized potential prey items into freshwater and marine prey and then divided them again into five isotopically clustered groups in similar taxonomic or trophic classes before fitting the Bayesian isotope mixing model: freshwater fish, freshwater invertebrates, marine fish, marine shrimps, and marine crabs. The trophic enrichment factors used in the model were 2.16 ± 0.35‰ (mean ± SD) for δ^13^C and 3.84 ± 0.26‰ for δ^15^N, which have been estimated for the feathers of birds [71]. We obtained the estimated proportion of marine and freshwater prey by posterior combination using the MixSIAR package in R software.

**Table 2:** δ^13^C and δ^15^N values (mean ± SD) of potential prey sources of Black-faced Spoonbill chicks collected in South Korea. Insect larvae in freshwater invertebrates were only identified to the family level.

Potential prey and origin		*n*	δ^13^C	δ^15^N	Reference
Freshwater					
Fish	*Pseudorasbora parva*	2	-26.2 ± 0.2	16.8 ± 0.4	[49]
	*Misgurnus* sp.	19	-26.77 ± 0.86	15.26 ± 1.31	[50]
Invertebrate	*Searma dehaani*	20	-25.18 ± 0.99	8.33 ± 0.93	[51]
	Coenagrionidae	8	-28.45 ± 0.35	7.07 ± 0.19	[52]
	Aeshnidae	24	-28.61 ± 0.38	5.65 ± 0.2	[52]
	Hydrophilidae	6	-27.09 ± 0.69	5.58 ± 0.8	[52]
	Dytiscidae	17	-28.1 ± 0.74	5.71 ± 0.31	[52]
Marine					
Fish	*Acanthogobius flavimanus*	2	-13.80 ± 0.33	15.7 ± 1.46	[53]
	*Amblychaeturichthys hexanema*	2	-14.61 ± 0.20	13.26 ± 0.80	[53]
	*Cryptocentrus filifer*	1	-14.68	14.64	[53]
	*Pseudoblennius cottoides*	1	-12.82	16.4	[53]
	*Mugil cephalus*	1	-14.9	13.86	[53]
Shrimp	*Alpheus brevicristatus*	1	-10.0	17.9	[54]
	*Metapenaeopsis dalei*	2	-10.2 ± 0.1	16.6 ± 0.5	[54]
Crab	*Ilyoplax pusilla*	5	-15.8 ± 1.0	11.7 ± 0.8	[55]
	*Macrophthalmus japonicus*	12	-11.9 ± 1.1	12.5 ± 1.1	[55]
	*Callianassa japonica*	6	-15.2 ± 2.4	12.2 ± 0.7	[55]

### Statistical analysis

We used a linear-mixed effect modeling method with maximum likelihood to determine age-dependent shifts and spatial variation in the diet of young Black-faced Spoonbills using the Lme4 [72] and lmerTest packages [73]. We fitted a linear-mixed effect model for each stable isotope with the same fixed and random effects. Individuals were included as random intercepts to account for variation among individuals because two portions of each feather were collected from each individual. In fixed effects, we included the chick-rearing period as a categorical variable (early: 10 days old vs. late: 22 days old) and breeding colony. The interaction term between the chick-rearing period and the breeding colony was included as a fixed effect because the degree of dietary shift of chicks might vary among the breeding populations.

Prey availability in rice paddies around Suhaam decreased from May to June due to the migration of fish and invertebrates during the mid-season drainage of rice paddies [36]. Mid-season drainage is common in rice cultivation in Korea; thus, we assumed that the seasonal pattern of prey availability might be similar between colonies. Therefore, we included hatching date as a covariate to account for temporal differences in foraging habitats that might influence the foraging behavior of spoonbills, such as prey availability. We also included the interaction between hatching date and breeding colony to account for spatial variation of phenology among breeding colonies and interaction between hatching date and chick-rearing period to examine whether there was a seasonal trend in the diet shift of chicks during the chick-rearing period.

To minimize the risk of type 1 error, we first tested the overall significance of the full model by comparing the full model with a null model with only an intercept and a random effect using a likelihood ratio test [74, 75]. We also tested the significance of the interaction term of the full model by comparing the full model with a reduced model lacking an interaction term to be tested using a likelihood ratio test. When interactions did not have a significant effect, we excluded them to allow for interpretation of the main effects [76]. We calculated the least-squares means with 95% confidence intervals for each level of the categorical variables, chick-rearing period, and breeding colony, and conducted post-hoc pairwise comparisons using Tukey’s test with the emmeans package [77]. We checked for heteroscedasticity and normality in the residuals of the model via visual inspection of the residual plots and did not find related issues. A p-value < 0.05 was considered statistically significant.

## Results

### Potential foraging habitats

Foraging habitat analysis indicated that three of four sites had both freshwater and marine foraging habitats within an 11-km radius. However, Gujido had limited areas for foraging (<8 km^2^) compared to the other colonies and had virtually no freshwater wetlands within the normal foraging range due to its remoteness from the nearest coastline (Fig 1B). The area of intertidal mudflats around the breeding colonies, except for Gujido, was two to six times larger than the freshwater wetlands (Fig 1B).

### δ^13^C and δ^15^N isotope values of chicks

**Fig 2:**
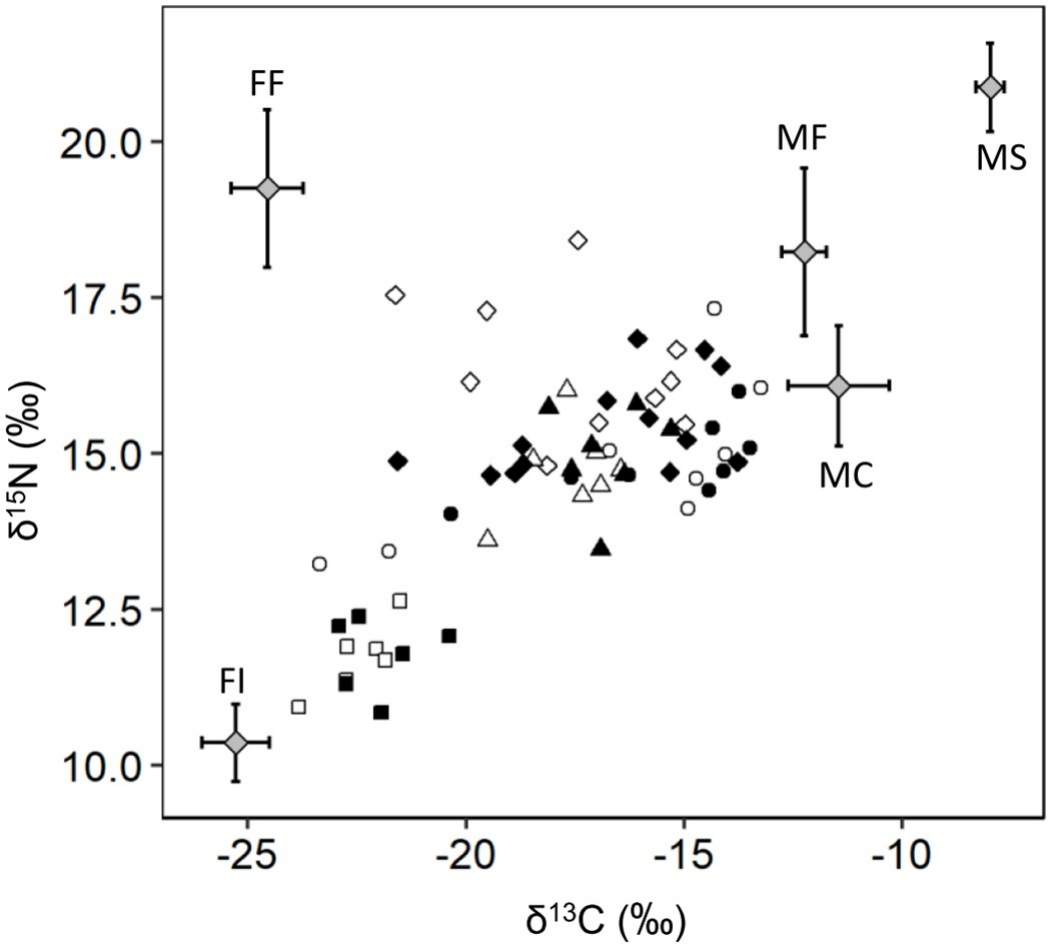
δ^13^C and δ^15^N values of Black-faced Spoonbill chicks and potential prey groups for diet reconstruction. The δ^13^C and δ^15^N values of the tip (early chick-rearing period; white) and middle (late chick-rearing period; black) portions of the primary feathers of Black-faced Spoonbill chicks at four breeding colonies: Gujido (square), Suhaam (circle), Namdongji (triangle), and Chilsando (diamond). Three individuals in the early chick-rearing period in Chilsando without δ^15^N values are not shown. Isotopic values of the five potential prey groups, corrected using the fractionation factor (71), are presented as gray diamonds: FF: freshwater fish, FI: freshwater invertebrate, MF: marine fish, MS: marine shrimp, and MC: marine crustacean.

The mean δ^13^C values of the early and late chick-rearing period were -18.17 ± 0.52‰ (mean and standard deviation, *n* = 34) and -17.42 ± 0.50‰ (*n* = 34), respectively (Fig 2). The mean δ^15^N value was 14.72 ± 0.34‰ (*n* = 31) for the early chick-rearing period and 14.55 ± 0.26‰ (*n* = 34) for the late chick-rearing period.

**Fig 3:**
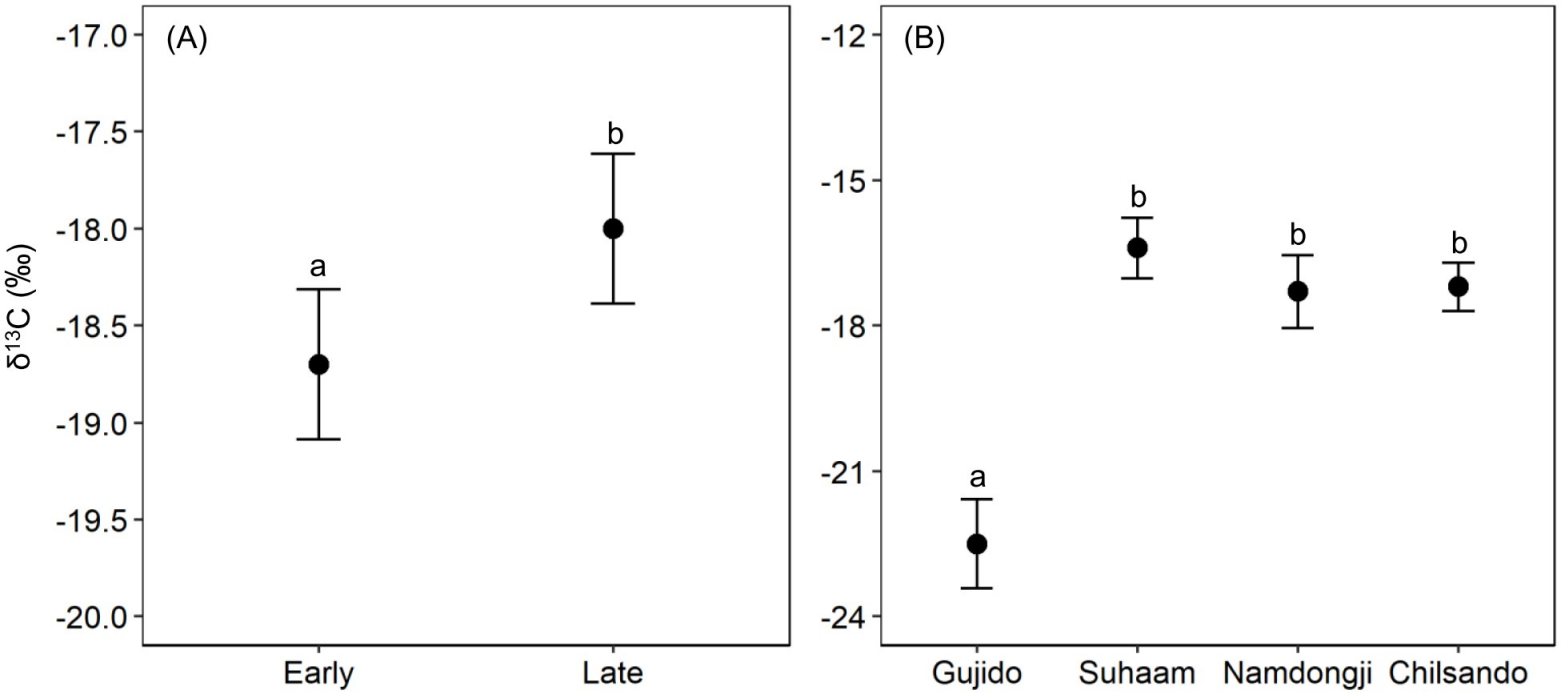
Least square means and standard error of the δ^13^C values of primary feathers of Black-faced Spoonbill chicks according to (A) chick-rearing period and (B) breeding colony. Means with the same letter are not significantly different (Tukey’s post hoc tests).

For δ^13^C analysis, the full model was significantly different from the null model (χ^2^ = 40.67, df = 9, p < 0.001). We found that there was a significant interaction effect between breeding colony and hatching date (breeding colony × hatching date: χ^2^ = 11.32, df = 3, p = 0.010) and removed other two-way interactions from the full model (chick-rearing period × breeding colony: χ^2^ = 2.14, df = 3, p = 0.544; chick-rearing period × hatching date: χ^2^ = 3.75, df = 1, p = 0.053; S1 Table). The reduced model showed a substantial difference in δ^13^C values between chick-rearing periods. δ^13^C values for the late chick-rearing period were higher 0.75 ± 0.29‰ (95% CI = 0.33–2.85; Fig 3A; S1 and S2 Figs) than those for the early chick-rearing period. δ^13^C values were substantially different among breeding colonies (Fig 3B). The least square means of δ^13^C values of chicks in Gujido (-22.5 ± 0.93‰) were lower than those of the other three colonies including Suhaam (-16.4 ± 0.62‰), Namdongji (-17.3 ± 0.75‰), and Chilsando (-17.2 ± 0.50‰; Fig 3B).

The interaction between breeding colony and hatching date indicated that the change in δ^13^C values with hatching timing varied among breeding colonies. Only in Suhaam, δ^13^C values of chicks increased 0.24 ± 0.07‰ per day (0.10–0.38‰/day) over the chick-rearing period, while there was no evidence of change on other colonies (Gujido: -0.04 ± 0.10‰/day -0.24–0.15‰/day; Namdongji: 0.02 ± 0.06‰/day -0.10–0.13‰/day; Chilsando: -0.05 ± 0.09‰/day, -0.23–0.12‰/day).

**Fig 4:**
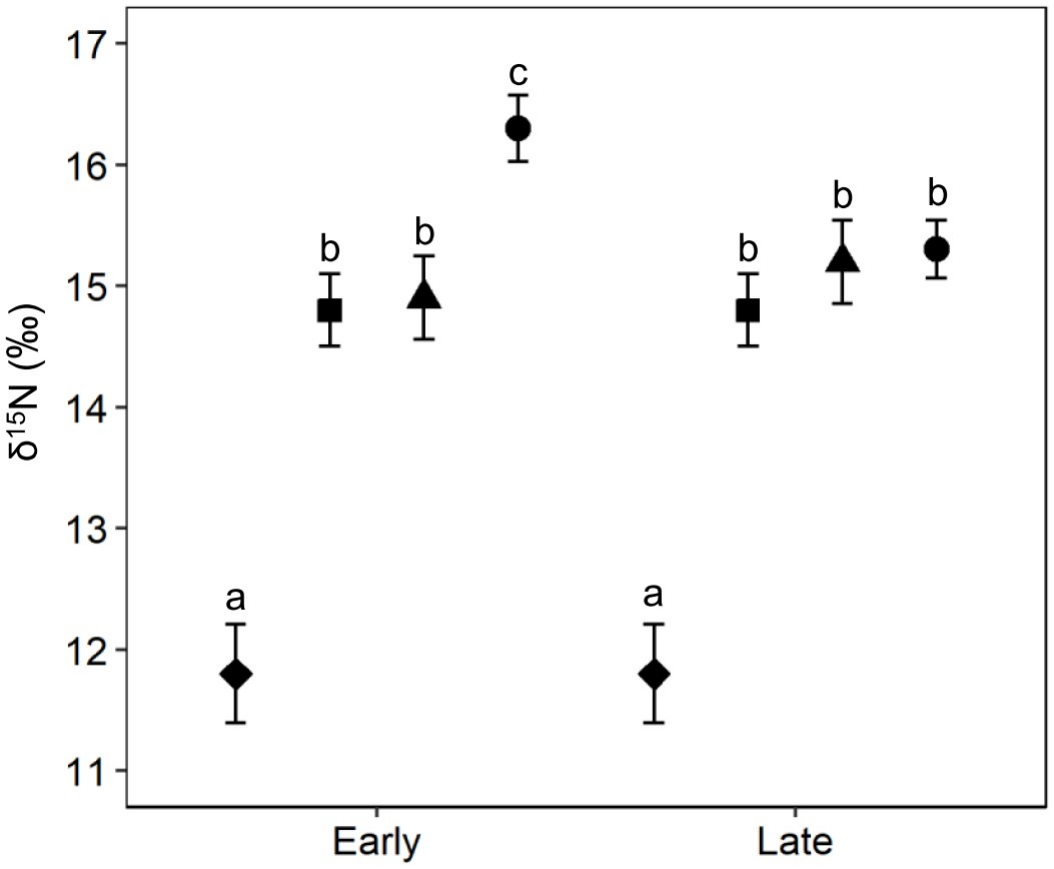
Least squares means and standard error of δ^15^N values of primary feathers of Black-faced Spoonbill chicks according to chick-rearing period and breeding colony. Means with the same letter are not significantly different (Tukey’s post hoc tests).

For δ^15^N, the full model differed significantly from the null model (χ^2^ = 80.13, df = 12, p<0.001). We found that there was a significant interaction between chick-rearing period and breeding colony (χ^2^ = 8.54, df = 3, p = 0.036) and breeding colony and hatching date (χ^2^ = 9.23, df = 3, p = 0.026), while the interaction between chick-rearing period and hatching date for δ^15^N was not significant (χ^2^ = 1.09, df = 1, p = 0.292; S2 Table). The reduced model showed that δ^15^N values of chicks differed between chick-rearing periods only in chicks at Chilsando, where δ^15^N values for the late chick-rearing period were 1.01 ± 0.33‰ lower (0.33–1.68‰) than those during the early chick-rearing period (Fig 4). There was no evidence of a difference in δ^15^N values between chick-rearing periods at other colonies (Gujido: -0.04 ± 0.45‰, -0.95–0.88‰; Suhaam: -0.01 ± 0.39‰, -0.81–0.77‰; Namdongji: -0.25 ± 0.42‰, -1.10–0.59‰; Fig 4). Variation in δ^15^N values among breeding colonies differed according to the chick-rearing period (Fig 4). For the early chick-rearing season, the feathers of chicks at Gujido had the lowest δ^15^N values (11.8 ± 0.41‰); among the other three colonies, the δ^15^N values of chicks at Chilsando (16.3 ± 0.41‰) were substantially higher than those at Suhaam (14.8 ± 0.30‰) and Namdonji (14.9 ± 0.34‰). For the late chick-rearing season, δ^15^N values of chicks were lower at Gujido (11.8 ± 0.41‰) than at other breeding colonies, and there was no difference among the other colonies (Suhaam: 14.8 ± 0.30‰, Namdonji: 15.2 ± 0.34‰, Chilsando: 15.3 ± 0.24‰; Fig 4).

We failed to find supportive evidence for the change in δ^15^N values by hatching date of chicks in any breeding colony (Gujido: 0.01 ± 0.07‰/day -0.06–0.09 ‰/day; Suhaam: 0.04 ± 0.03‰/day, -0.01–0.09 ‰/day; Namdongji: -0.03 ± 0.02‰/day, -0.07–0.01 ‰/day; Chilsando: -0.06 ± 0.03‰/day, -0.13–0.01 ‰/day).

### Estimated proportion of diet by chick-rearing period and breeding colony

**Fig 5:**
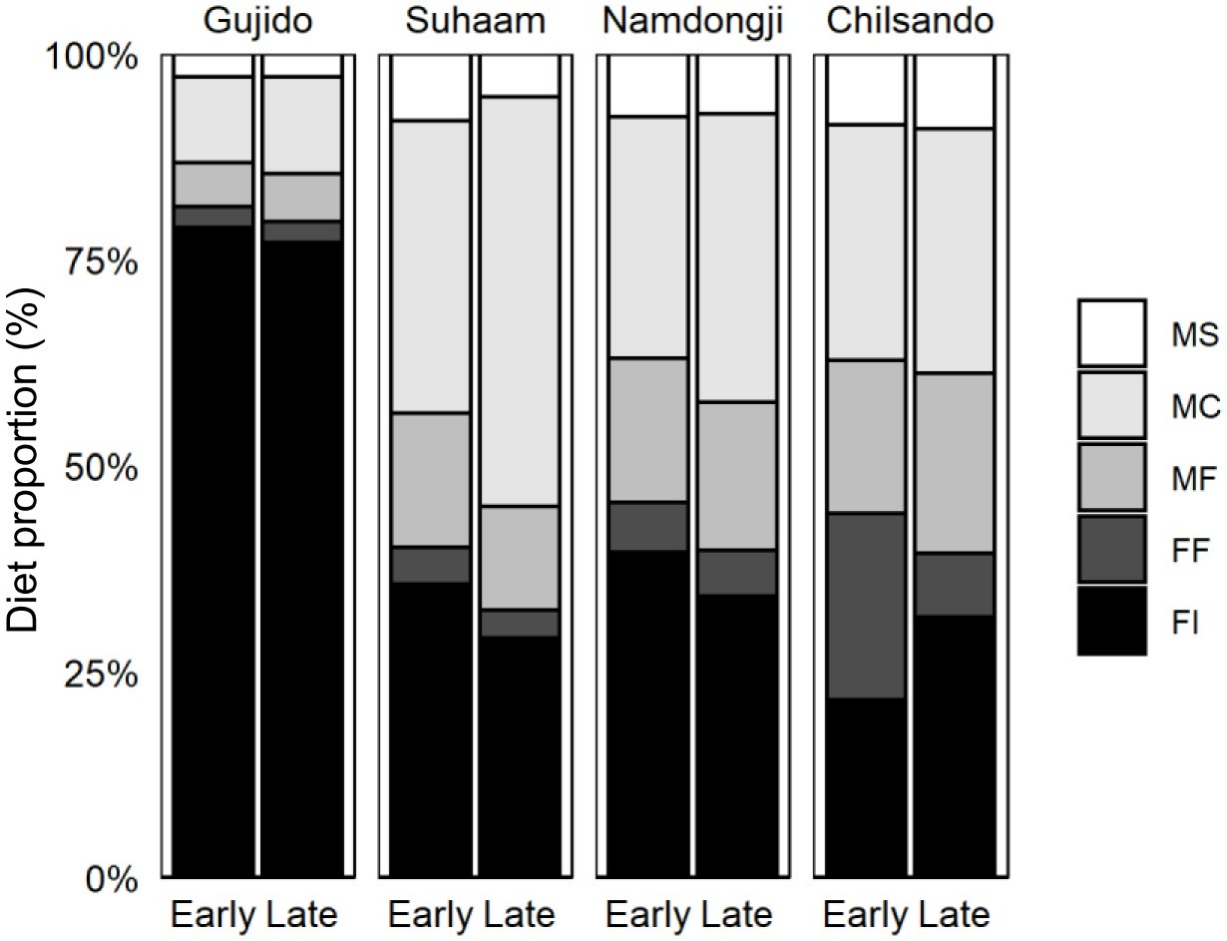
Estimated proportion of freshwater and marine prey in the diet of Black-faced Spoonbills at four breeding colonies during two chick-rearing periods. FF: freshwater fish, FI: freshwater invertebrate, MF: marine fish, MS: marine shrimp, and MC: marine crustacean.

The Bayesian mixing model also showed a spatial difference in diet use between breeding colonies (Fig 5), which indicated that the diet composition of spoonbill chicks at Gujido was mostly different from that in other breeding colonies. Despite freshwater wetlands not being available for foraging in the vicinity (Fig 1, Table 1), chicks at Gujido were fed almost entirely freshwater diets (>79%) in both age groups, and these diets were dominated by freshwater invertebrates (Fig 5). On the other hand, other populations that could access freshwater wetlands and intertidal areas used both marine and freshwater food sources at a similar rate during the chick-rearing period (Fig 5). The model showed that chicks at Chilsando were fed with more freshwater and marine fish during the early post-hatching period than other colonies (Fig 5). On the other hand, there was high overlap of estimated diet contributions between the early and late chick-rearing periods (Fig 5).

## Discussion

The most prominent finding was that the proportion of freshwater diets was high at Gujido, where no freshwater wetlands are available for foraging within the typical foraging range (11 km from the colony). On the other hand, at other colonies located near the coastline, including Suhaam, Namdongji, and Chilsando, adults fed their chicks with prey from intertidal mudflats and freshwater wetlands at a similar rate. This result is in agreement with those of previous reports, which showed that the adults at other offshore islands did not feed in nearby intertidal flats but from more distant freshwater wetlands instead, while those at inshore islands used both habitats. These results also corroborate the findings of previous reports showing that the diets of chicks in offshore colonies mainly originated from freshwater habitats [17, 18]. With regard to temporal change in the proportion of freshwater diets, our study showed that δ^13^C values, which were used to examine diet proportions from freshwater and intertidal wetlands, were higher during the early chick-rearing period than during the late chick-rearing period. In addition, δ^13^C values for Suhaam increased along with hatching timing. These results indicate that, in Suhaam, the freshwater diet proportion of chicks might be higher during the early chick-rearing season than the late chick-rearing period, while early-breeding spoonbills might feed their chicks with more freshwater prey than spoonbills breeding later. These results are consistent with earlier observations conducted in the coastal areas around Suhaam, which showed that the number of spoonbills foraging in rice paddies decreased over the chick-rearing season in the coastal area around Suhaam [17, 36, 78].

Our data could not conclusively prove or disprove the hypothesis presented in previous reports that adults may feed a larger freshwater diet proportion during the early chick-rearing period due to lower salt tolerance among young chicks. To definitively support the salt stress hypothesis with our stable isotope analysis, δ^13^C values and the estimated proportion of freshwater prey should have shown lower salt intake or exclusive use of freshwater prey during the early chick-growing period. However, the range of δ^13^C demonstrated high overlap between the early and late chick-rearing periods, and some of the individuals at Suhaam, Namdongji, and Chilsando might have been fed with more marine prey than freshwater prey even during the early chick-rearing period. δ^13^C and δ^15^N Bayesian mixing models estimated that more than 50% of diets during the early chick-rearing period originated from intertidal mudflats at Suhaam, Namdongji, and Chilsando. In addition, the contribution of each prey type did not vary substantially by chick-rearing period. Comparing the estimated contribution of diets between chick-rearing periods may be limited because we estimated the average contribution of each prey type by colony and chick-rearing period without accounting for within-individual variation in δ^13^C and δ^15^N. In addition, we used potential prey sources in the model, not data collected at foraging sites of study populations, which might influence the uncertainty of the estimation. Nonetheless, δ^13^C values indicated that salt intake might not be substantially different between chick-rearing periods, suggesting a sufficient osmoregulatory capacity to consume marine diets among chicks during the early growing period.

However, age-related changes and spatial variation in δ^13^C from our analysis indicate that the hypothesis of osmoregulatory system development should be investigated by further research. For the Laughing Gull, chicks exhibit lower salt tolerance than adults, and prey exceeding a certain level of salinity negatively affect chick growth rate [24]. Stable isotope analysis showed that breeding gulls on inshore islands delivered a substantially higher proportion of terrestrial prey during the early chick-growing stage than during the pre-fledgling stage [27]. However, even during the early chick-growing period, they fed both terrestrial insects and marine prey, and δ^13^C values of some individuals were close to marine invertebrates, which are osmo-conformers with body fluid concentrations similar to seawater [79]. This result indicates that feeding low salt prey and marine prey together may be more advantageous for mitigating salt stress on chicks than feeding only marine prey if chicks do not have fully developed osmoregulatory systems. In our results, although the δ^13^C values of young spoonbills increased slightly with age, the shift in δ^13^C values regardless of breeding season and colony might partially reflect a change in the proportion of freshwater and marine diet during the chick-rearing period. Previous studies showed that the salt glands of chicks become functional around six days after hatching in many waterbirds [80–82]. Thus, chicks whose primary feathers are beginning to grow might have partially or even fully functioning osmoregulatory systems and be fed with marine prey, as reflected by the tip portion of the primary feathers. δ^13^C and δ^15^N values in the growing feathers gradually change along the shaft after the dietary shift [83]. Therefore, the stable isotopes in the two portions of the feathers might not fully represent the dietary shifts of chicks due to the dilution effect during the isotopic assimilation process. To better understand the importance of low salt diets in newborn chicks, further experimental and physiological analyses of the diets of chicks during the early hatching period (1–5 days) should be conducted using blood plasma, which has a half-life of only 3 days [84, 85], in addition to anatomical studies.

Physiological factors such as nutrition status and growth may influence the stable isotope ratio of chicks [86–88]. Previous studies showed that these factors mainly influence δ^15^N values due to changes in nitrogen use efficiency depending on nutrition status [86, 87]. For Blue-footed Booby chicks, feather δ^13^C values were negatively correlated with body condition [89]. However, a linear mixed model of δ^13^C values accounted for individual variation concerning nutritional intake and body condition, showing a consistent shift in δ^13^C values during the chick-rearing period. Therefore, these results suggest that variation in δ^13^C values with the growth of chicks might not relate to physiological factors but instead to changes in diet composition.

The proportion of freshwater prey of chicks in each colony also showed the possibility that young spoonbills might be more susceptible to salt intake than adults. All breeding populations used freshwater wetlands to a considerable degree, despite the availability of marine resources in intertidal mudflats located closer to colonies. The central place foraging theory predicts that a forager will favor large-sized or high-quality prey to maximize the net rate of energy delivery as foraging distance increases [1]. In the context of this theory, the use of intertidal mudflats with high availability and accessibility might be more effective for reducing energy expenditure of spoonbills during foraging. Nevertheless, foraging in freshwater wetlands indicated that freshwater diets might be more energetically profitable because of the high expense of metabolic energy for the secretion of excess salt, even in chicks with functioning salt glands [89]. The high proportion of freshwater diets at offshore islands, including Gujido in this study, was similar to White Ibises at a coastal colony, which showed that adults rearing chicks concentrated their foraging efforts in freshwater wetlands [30]. Observational and experimental studies of White Ibises showed that feeding on invertebrates from salt marshes severely affected the survival and growth of chicks [23, 30, 90]. Oceanographic studies of the west coast of South Korea showed that the seawater around Gujido had a salinity falling within the euhaline range (30–35 ppt) during the entire year [91–93]. On the other hand, the salinity of coastal waters around Suhaam and Namdongji was in the polyhaline (18–30 ppt) or mesohaline (5–18 ppt) range due to the increasing inflow of freshwater in spring and summer [92]. The results suggest that prey from intertidal habitats might cause higher saline stress on chicks at Gujido than those in other colonies in inshore areas. Thus, the chicks at Gujido might need more freshwater prey to mitigate the energy expense of the osmoregulation process when consuming marine prey. Although there have been no studies on the effect of foraging habitat salinity on diet selection in waterbirds with lower salt tolerance among chicks, a previous study showed that salt intake above a specific concentration significantly affected the growth of waterbird chicks [27]. In addition, due to the long distance to freshwater wetlands from Gujido, which was at least 15 km away, spoonbills in Gujido might deliver more freshwater prey at one time to compensate for decreased feeding rates. However, this explanation is limited to speculation because we had no detailed information on foraging environments, including prey availability, and the sample size was small. Thus, future research should investigate the effects of salt stress on the growth and body condition of young spoonbills according to the salinity of prey and the chick-growing period.

δ^13^C values at Suhaam showed that chicks hatched later were fed more marine prey. This seasonal change in the proportion of freshwater diets in Suhaam might be related to the decrease in prey availability at rice paddies over the breeding season due to mid-season drainage. On the other hand, there was no significant seasonal trend in δ^13^C values at Gujido, Namdongji, and Chilsando. Although there has been no detailed investigation of the foraging habitats around breeding colonies except for Suhaam, a few observations reported that spoonbills at Namdongji foraged in different types of freshwater wetlands such as reservoirs and lakes [48]. Therefore, these differences are likely related to variation in reliance on rice paddies according to breeding colonies. However, we did not systematically collect feathers over the chick-rearing season, and the range in hatching dates of chicks differed among breeding colonies. Therefore, to determine the effect of seasonal changes in foraging environments, the prey source of chicks at different hatching times needs to be analyzed using systematic sampling.

The average trophic level of chick diet, estimated by δ^15^N values, at Gujido was lower than those of other three populations, while those at Chilsando were higher than those at Suhaam and Namdongji only during the early chick-rearing period. These results were consistent with the high estimated diet proportion of freshwater invertebrates at the lowest trophic level at Gujido, while chicks at Chilsando were fed more freshwater and marine fish at higher trophic levels than youngs at Suhaam and Namdongji in the early chick-rearing period. Although δ^15^N values at Chilsando decreased with age between the early and late chick-growing periods, there was no significant and consistent change in δ^15^N values over time and the values varied among individuals at Gujido, Suhaam, and Namdongji (S3 Fig). In other *Platalea* species that forage in freshwater or marine wetlands during the breeding season, the diet composition of fish and invertebrates shows high inter- and intra-annual and spatial variation with the availability of prey in foraging habitats [3, 93]. Therefore, differences in the availability of prey around breeding colonies might lead to inconsistent variation in the proportion of fish and invertebrates or crustaceans in the diets of chicks within and among populations.

Our study found that δ^13^C values of young Black-faced Spoonbills enriched as they grew, showing that the proportion of freshwater diet might be lower during the late chick-rearing period than the early chick-rearing period. However, the slight change in δ^13^C values was not enough to support the hypothesis of a developing osmoregulatory system because it indicated that the salt intake of chicks does not appear to vary dramatically between the early and late growing periods. Further work is required to establish whether this increase of δ^13^C values partially reflects a decrease in the proportion of freshwater diet along with the development of osmoregulatory ability. Future research should investigate diet contribution in the early-growth and pre-fledgling stages regarding prey availability in foraging habitats around study populations. Nonetheless, we found that chicks in all populations consumed a significant proportion of freshwater prey even at sites where intertidal mudflats were more accessible than freshwater wetlands. In addition, populations at more offshore colonies (for instance, Gujido in this study) showed no inflow of freshwater to nearby intertidal habitats and showed a heavy reliance on freshwater prey. These findings emphasize that the availability of freshwater wetlands may be beneficial to Black-faced Spoonbills strictly breeding in marine environments, as shown in the case of White Ibises breeding in South Carolina [23, 30].

Freshwater marshes, river estuaries, and intertidal mudflats on the western Korean Peninsula, which are potential foraging habitats for Black-faced Spoonbills, have steeply declined over the last 50 years due to reclamation, construction of river barrages and seawalls, and coastal development [12, 94]. Approximately 60% of the tidal flat area of the Yellow Sea region of the Korean Peninsula has been lost [12], and the estuarine wetland area in South Korea decreased by 90% from the 1970s to the 2000s [94]. Likewise, approximately 44% of rice paddies, which are important artificial wetlands for spoonbill foraging, also disappeared between 1989 and 2017 in South Korea due to the conversion of rice paddies into farmland and greenhouse facilities for high-value crop production [94–97]. In the coastal region of the Gyeonggi Province, where most breeding colonies of spoonbills are located, the loss of foraging habitat is much more severe due to the expansion of metropolitan areas [98, 99]. In addition to the decline in foraging habitat area, heavy use of agrochemicals is likely to have reduced the diversity and abundance of potential prey, such as fish and invertebrates, in rice paddies and around wetlands [100–102].

Our findings regarding freshwater diet reliance in spoonbill chicks suggest that the ongoing loss of quantity and quality of coastal freshwater wetlands in the vicinity of existing breeding colonies may result in greater energy expenditure for both provisioning adults and growing chicks. This expenditure may negatively affect the reproductive output of spoonbills adapted to marine breeding habitats, such as spoonbills breeding in offshore colonies, due to the high freshwater diet requirements of their chicks. Protection of coastal freshwater wetlands appears to be as important as the preservation of intertidal mudflats in the conservation of endangered Black-faced Spoonbills. Consequently, retention and better management of natural freshwater wetlands and intertidal mudflats as well as sound agricultural practices in rice paddies could help to improve the breeding success of Black-faced Spoonbills.

## Supporting information

S1 Fig δ^13^C values of Black-faced Spoonbill chicks along with growth in four breeding colonies. (A) Gujido, (B) Suhaam, (C) Namdongji, and (D) Chilsando. Lines link the δ^13^C values for each individual between chick-rearing periods.
(TIF)

S2 Fig Age-dependent changes in δ^13^C values of Black-faced Spoonbill chicks over the breeding season. Hatching dates were adjusted for each breeding colony by subtracting the hatching dates of individuals from the earliest hatching dates. The solid line and grey shading represent the estimated mean and standard error of effect size for age in the linear-mixed effect model for δ^13^C values.
(TIF)

S3 Fig δ^15^N values of Black-faced Spoonbill chicks along with growth in four breeding colonies. (A) Gujido, (B) Suhaam, (C) Namdongji, and (D) Chilsando. Lines link the δ^15^N values for each individual between chick-rearing periods.
(TIF)

S1 Table Full and reduced linear mixed-effects model of δ^13^C values from primary feathers of Black-faced Spoonbill chicks. The model included individual ID as a random effect. The intercept in this model estimated δ^13^C values in the early chick-rearing period at the Gujido colony.
(DOCX)

S2 Table Full and reduced linear mixed-effects model of δ^15^N values from primary feathers of Black-faced Spoonbill chicks. The model included individual ID as a random effect. The intercept in this model estimated δ^15^N values in the early chick-rearing period at the Gujido colony.
(DOCX)

S3 Table δ^13^C and δ^15^N values from primary feathers of Black-faced Spoonbill chicks. (XLSX)
