# Supplementary figures and images for: Age-dependent shifts and spatial variation in the diet of endangered Black-faced Spoonbill (Platalea minor) chicks
Source: PLoS One. 2021 Jul 9;16(7):e0253469. doi: 10.1371/journal.pone.0253469 (PMC8270140; doi:10.1371/journal.pone.0253469)

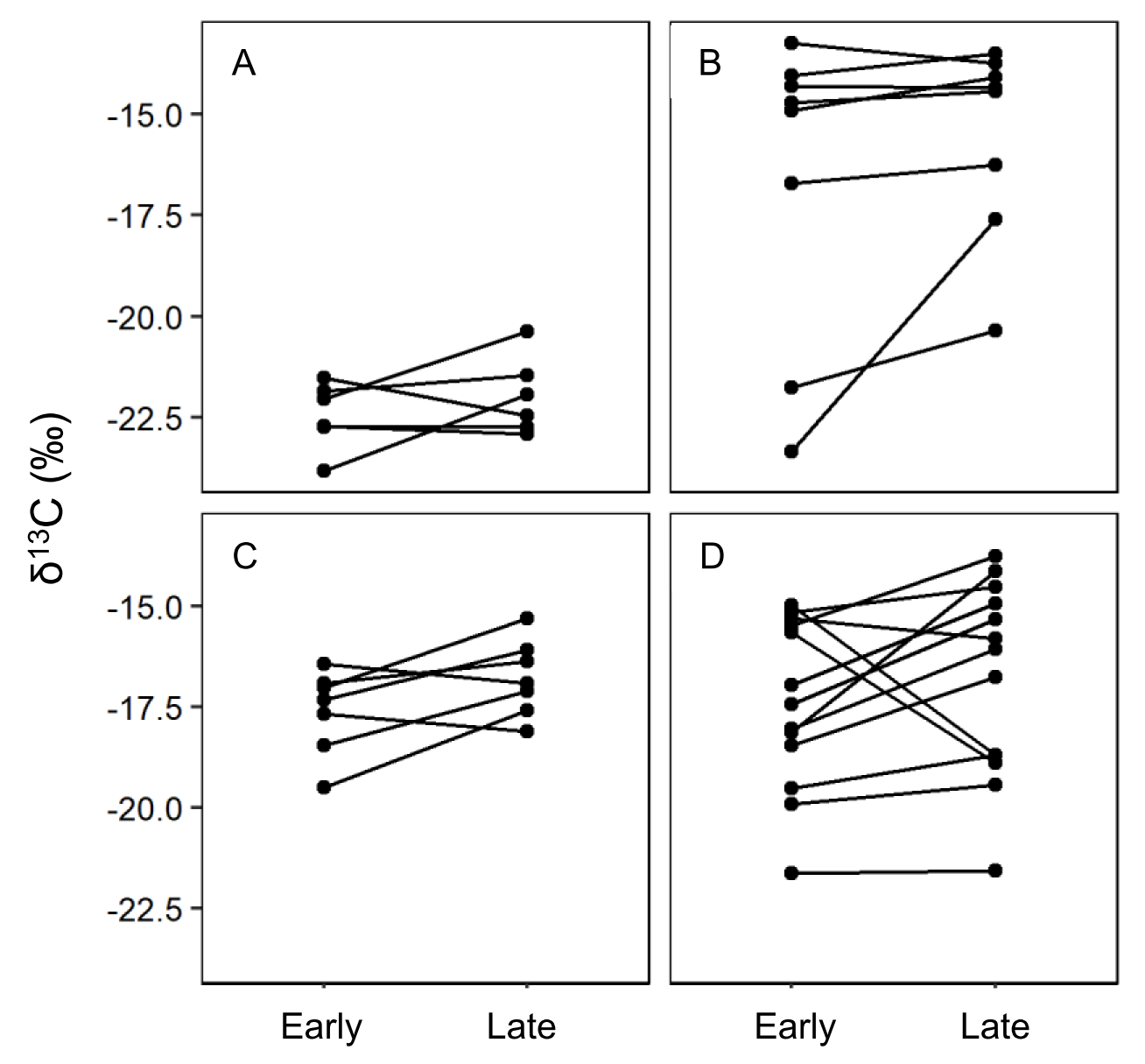

Supplement: S1 Fig — (A) Gujido, (B) Suhaam, (C) Namdongji, and (D) Chilsando. Lines link the δ13C values for each individual between chick-rearing periods. (TIF) [file pone.0253469.s001.tif]

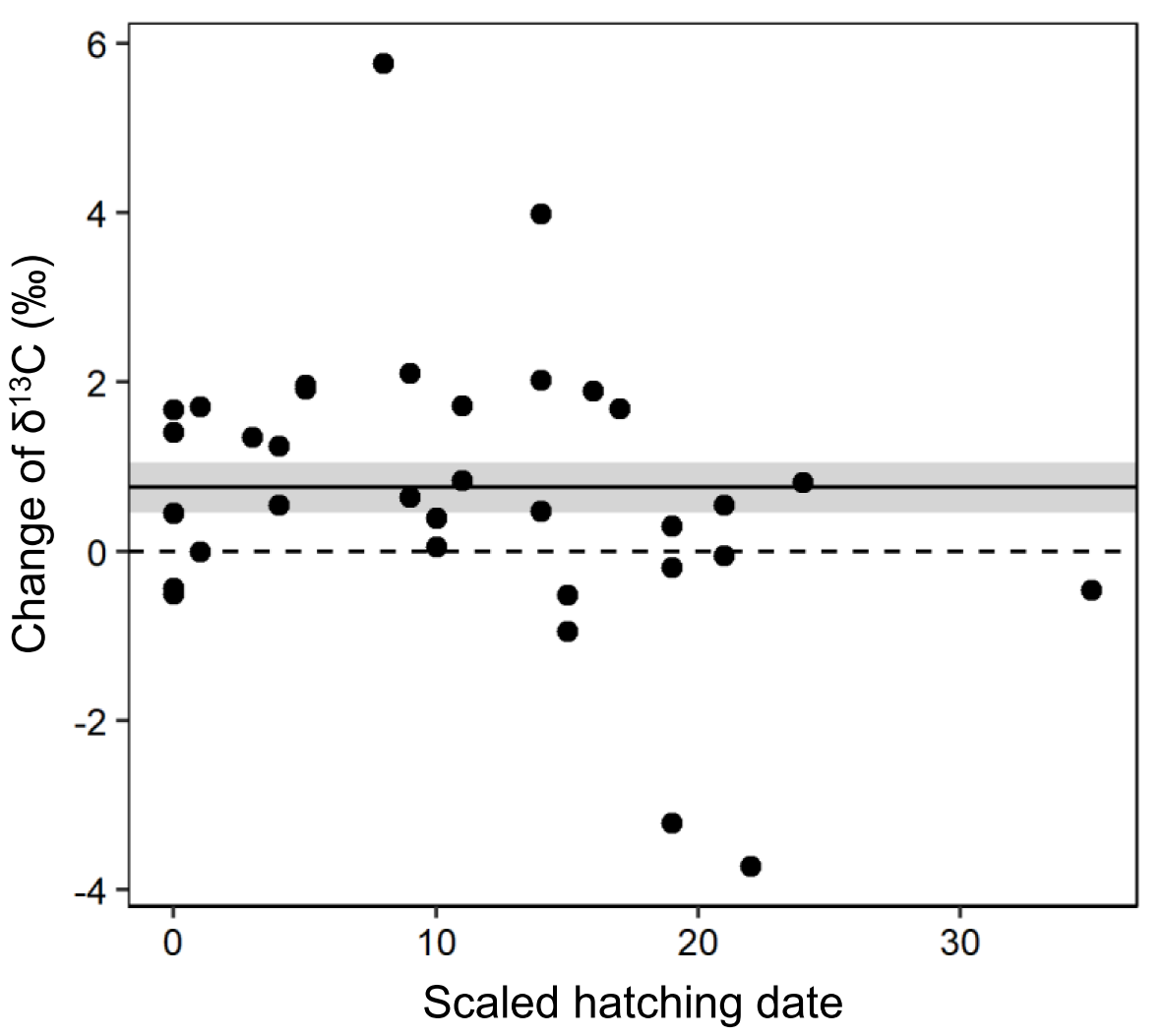

Supplement: S2 Fig — Hatching dates were adjusted for each breeding colony by subtracting the hatching dates of individuals from the earliest hatching dates. The solid line and grey shading represent the estimated mean and standard error of effect size for age in the linear-mixed effect model for δ13C values. (TIF) [file pone.0253469.s002.tif]

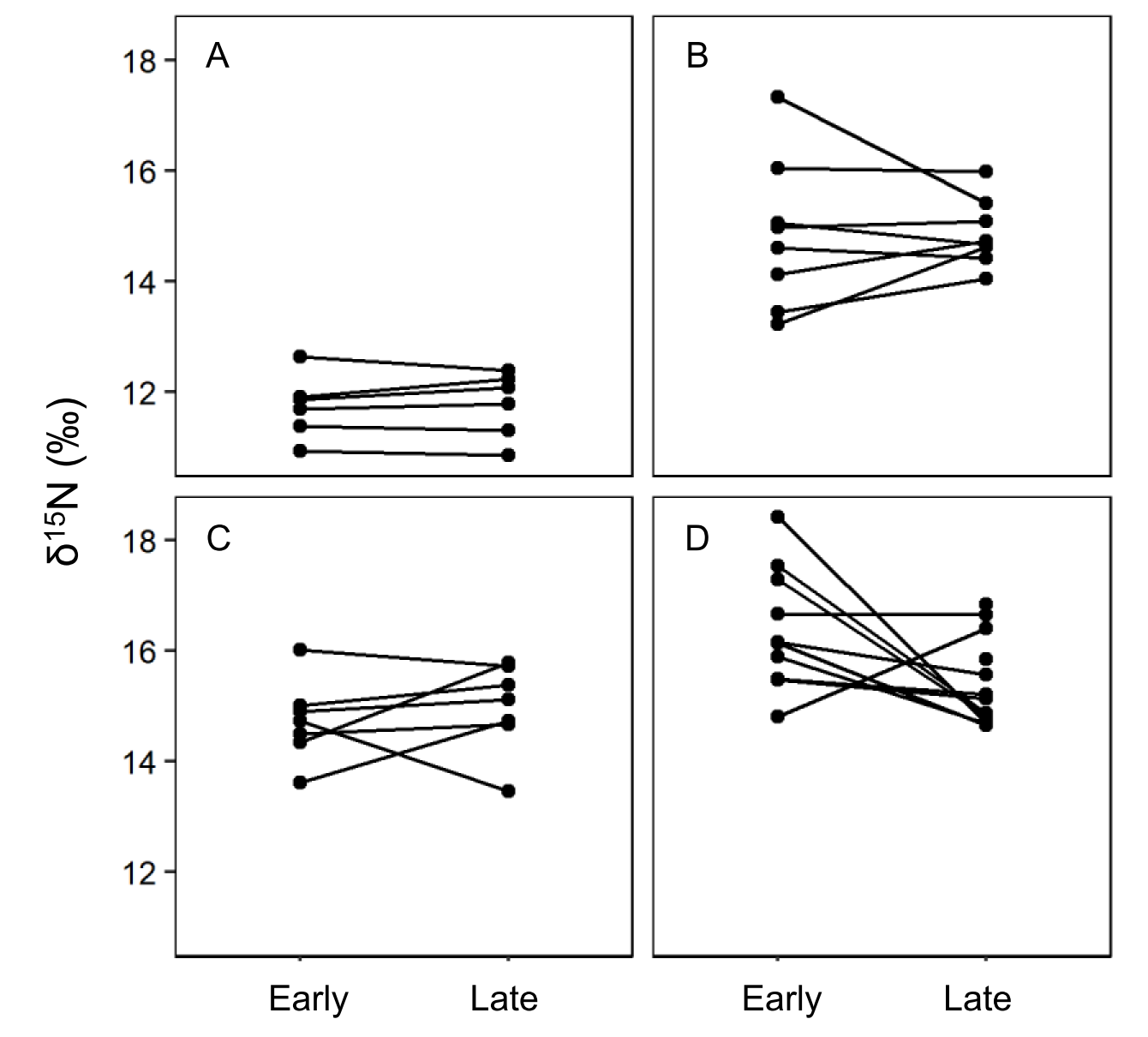

Supplement: S3 Fig — (A) Gujido, (B) Suhaam, (C) Namdongji, and (D) Chilsando. Lines link the δ15N values for each individual between chick-rearing periods. (TIF) [file pone.0253469.s003.tif]
