# Supplementary material for: Age-dependent shifts and spatial variation in the diet of endangered Black-faced Spoonbill (Platalea minor) chicks
Source: PLoS One. 2021 Jul 9;16(7):e0253469. doi: 10.1371/journal.pone.0253469 (PMC8270140; doi:10.1371/journal.pone.0253469)
Supplement: S1 Table — The model included individual ID as a random effect. The intercept in this model estimated δ13C values in the early chick-rearing period at the Gujido colony. (DOCX) [file pone.0253469.s004.docx]

# Age-dependent shifts and spatial variation in the diet of endangered Black-faced Spoonbill (*Platalea minor*) chicks

**Min-Su Jeong^1^, Chang-Young Choi^1,2^*, Woo-Shin Lee^1,2^, Ki-Sup Lee^3^**

**DOI: 10.1371/journal.pone.0253469**

**S1 Table. Full and reduced linear mixed-effects model of δ^13^C values from primary feathers of Black-faced Spoonbill chicks.** The model included individual ID as a random effect. The intercept in this model estimated δ^13^C values in the early chick-rearing period and in the Gujido colony.

| **Fixed effects** | **Estimates** | **SE** | **Confidence interval** | **t** | **p** |
| --- | --- | --- | --- | --- | --- |
| **Full model** |  |  |  |  |  |
| Intercept | -22.35 | 1.27 | -24.89 – -19.81 | -17.66 | <0.001 |
| Chick-rearing period Late | 1.27 | 0.76 | -0.26 – 2.80 | 1.67 | 0.103 |
| Breeding colony Suhaam | 0.40 | 1.82 | -3.26 – 4.05 | 0.22 | 0.829 |
| Breeding colony Namdongji | 3.51 | 1.89 | -0.28 – 7.30 | 1.86 | 0.070 |
| Breeding colony Chilsando | 5.25 | 1.86 | 1.51 – 8.99 | 2.83 | 0.008 |
| Hatching date | -0.01 | 0.09 | -0.18 – 0.17 | -0.11 | 0.917 |
| Chick-rearing period Late × Breeding colony Suhaam | 1.10 | 0.88 | -0.68 – 2.89 | 1.25 | 0.221 |
| Chick-rearing period Late × Breeding colony Namdongji | 1.17 | 0.96 | -0.77 – 3.12 | 1.22 | 0.232 |
| Chick-rearing period Late × Breeding colony Chilsando | 0.46 | 0.79 | -1.14 – 2.06 | 0.58 | 0.564 |
| Chick-rearing period Late × Hatching date | -0.07 | 0.03 | -0.13 – 0.00 | -1.99 | 0.055 |
| Breeding colony Suhaam × Hatching date | 0.28 | 0.10 | 0.07 – 0.49 | 2.71 | 0.010 |
| Breeding colony Namdongji × Hatching date | 0.06 | 0.10 | -0.14 – 0.26 | 0.59 | 0.557 |
| Breeding colony Chilsando × Hatching date | -0.01 | 0.11 | -0.24 – 0.22 | -0.10 | 0.922 |
| **Reduced model** |  |  |  |  |  |
| Intercept | -22.09 | 1.2 | -24.54 – -19.64 | -18.17 | <0.001 |
| Chick-rearing period Late | 0.75 | 0.29 | 0.17 – 1.33 | 2.60 | 0.014 |
| Breeding colony Suhaam | 0.95 | 1.76 | -2.61 – 4.51 | 0.54 | 0.595 |
| Breeding colony Namdongji | 4.10 | 1.82 | 0.42 – 7.78 | 2.25 | 0.031 |
| Breeding colony Chilsando | 5.48 | 1.82 | 1.82 – 9.48 | 3.02 | 0.005 |
| Hatching date | -0.04 | 0.08 | -0.21 – 0.13 | -0.49 | 0.625 |
| Breeding colony Suhaam × Hatching date | 0.28 | 0.10 | 0.07 – 0.49 | 2.71 | 0.010 |
| Breeding colony Namdongji × Hatching date | 0.06 | 0.10 | -0.14 – 0.26 | 0.59 | 0.557 |
| Breeding colony Chilsando × Hatching date | -0.01 | 0.11 | -0.24 – 0.22 | -0.10 | 0.922 |
