# Supplementary material for: Age-dependent shifts and spatial variation in the diet of endangered Black-faced Spoonbill (Platalea minor) chicks
Source: PLoS One. 2021 Jul 9;16(7):e0253469. doi: 10.1371/journal.pone.0253469 (PMC8270140; doi:10.1371/journal.pone.0253469)
Supplement: S2 Table — The model included individual ID as a random effect. The intercept in this model estimated δ15N values in the early chick-rearing period at the Gujido colony. (DOCX) [file pone.0253469.s005.docx]

# Age-dependent shifts and spatial variation in the diet of endangered Black-faced Spoonbill (*Platalea minor*) chicks

**Min-Su Jeong^1^, Chang-Young Choi^1,2^*, Woo-Shin Lee^1,2^, Ki-Sup Lee^3^**

**DOI: 10.1371/journal.pone.0253469**

**S2 Table. Full and reduced linear mixed-effects model of δ^15^N values from primary feathers of Black-faced Spoonbill chicks.** The model included individual ID as a random effect. The intercept in this model estimated δ^15^N values in the early chick-rearing period and in the Gujido colony.

| **Fixed effects** | **Estimates** | **SE** | **Confidence interval** | **t** | **p** |
| --- | --- | --- | --- | --- | --- |
| **Full model** |  |  |  |  |  |
| Intercept | 11.62 | 0.49 | 10.65 – 12.60 | 23.94 | <0.001 |
| Chick-rearing period Late | 0.04 | 0.42 | -0.81 – 0.89 | 0.09 | 0.928 |
| Breeding colony Suhaam | 2.38 | 0.70 | 0.98 – 3.78 | 3.41 | 0.001 |
| Breeding colony Namdongji | 3.90 | 0.72 | 2.46 – 5.35 | 5.41 | <0.001 |
| Breeding colony Chilsando | 5.78 | 0.73 | 4.31 – 7.26 | 7.87 | <0.001 |
| Hatching date | 0.01 | 0.03 | -0.05 – 0.07 | 0.30 | 0.764 |
| Chick-rearing period Late × Breeding colony Suhaam | -0.02 | 0.56 | -1.15 – 1.11 | -0.04 | 0.972 |
| Chick-rearing period Late × Breeding colony Namdongji | 0.21 | 0.57 | -0.95 – 1.38 | 0.37 | 0.712 |
| Chick-rearing period Late × Breeding colony Chilsando | -1.04 | 0.52 | -2.09 – 0.02 | -2.00 | 0.054 |
| Chick-rearing period Late × Hatching date | 0.03 | 0.04 | -0.04 – 0.11 | 0.91 | 0.372 |
| Breeding colony Suhaam × Hatching date | -0.04 | 0.04 | -0.11 – 0.03 | -1.16 | 0.253 |
| Breeding colony Namdongji × Hatching date | -0.07 | 0.04 | -0.15 – 0.02 | -1.60 | 0.118 |
| Breeding coloniy Chilsando × Hatching date | -0.02 | 0.56 | -1.15 – 1.11 | -0.04 | 0.972 |
| **Reduced model** |  |  |  |  |  |
| Intercept | 11.62 | 0.49 | 10.65 – 12.60 | 23.94 | <0.001 |
| Chick-rearing period Late | 0.04 | 0.42 | -0.81 – 0.89 | 0.09 | 0.928 |
| Breeding colony Suhaam | 2.38 | 0.70 | 0.98 – 3.78 | 3.41 | 0.001 |
| Breeding colony Namdongji | 3.90 | 0.72 | 2.46 – 5.35 | 5.41 | <0.001 |
| Breeding colony Chilsando | 5.78 | 0.73 | 4.31 – 7.26 | 7.87 | <0.001 |
| Hatching date | 0.01 | 0.03 | -0.05 – 0.07 | 0.30 | 0.764 |
| Chick-rearing period Late × Breeding colony Suhaam | -0.02 | 0.56 | -1.15 – 1.11 | -0.04 | 0.972 |
| Chick-rearing period Late × Breeding colony Namdongji | 0.21 | 0.57 | -0.95 – 1.38 | 0.37 | 0.712 |
| Chick-rearing period Late × Breeding colony Chilsando | -1.04 | 0.52 | -2.09 – 0.02 | -2.00 | 0.054 |
| Breeding colony Suhaam × Hatching date | 0.03 | 0.04 | -0.04 – 0.11 | 0.91 | 0.372 |
| Breeding colony Namdongji × Hatching date | -0.04 | 0.04 | -0.11 – 0.03 | -1.16 | 0.253 |
| Breeding colony Chilsando × Hatching date | -0.07 | 0.04 | -0.15 – 0.02 | -1.60 | 0.118 |
